# Supplementary material for: Facioscapulohumeral Muscular Dystrophy is Associated With Altered Myoblast Proteome Dynamics
Source: Mol Cell Proteomics. 2023 Jun 22;22(8):100605. doi: 10.1016/j.mcpro.2023.100605 (PMC10392138; doi:10.1016/j.mcpro.2023.100605)
Supplement: Supplemental Figures S1–S5 [file mmc1.pdf]

Supplemental Information

Facioscapulohumeral muscular dystrophy is associated with altered myoblast proteome dynamics

Yusuke Nishimura[1*], Adam J. Bittel[2*], Connor A. Stead[1], Yi-Wen Chen[2†¥] and Jatin G Burniston[1†§]

[1]Research Institute for Sport & Exercise Sciences, Liverpool John Moores University, Liverpool, L3 3AF, United Kingdom. [2]Center for Genetic Medicine Research, Children's National Medical Center, Washington, DC, USA.

[*]Joint First Authors

[†]Joint Senior Authors

[¥]Address for Correspondence: Yi-Wen Chen, Center for Genetic Medicine Research, Children's National Hospital, Washington, DC, 20010, USA. Email: ychen@childrensnational.org

[§]Address for Correspondence: Professor Jatin G Burniston. Research Institute for Sport & Exercise Sciences (RISES), Liverpool Centre for Cardiovascular Science (LCCS), Liverpool John Moores University, Tom Reilly Building, Byrom Street, Liverpool, L3 3AF, United Kingdom. Tel: +44 (0) 151 904 6265 Email: j.burniston@ljmu.ac.uk

Running title: Dynamic proteome profiling of myoblasts from individuals affected by FSHD

Keywords:
FSHD; deuterium oxide; heavy water; fractional synthesis rate; biosynthetic labelling; protein turnover; skeletal muscle; proteome dynamics; mitochondria; mitochondrial ribosome

Supplemental Figures

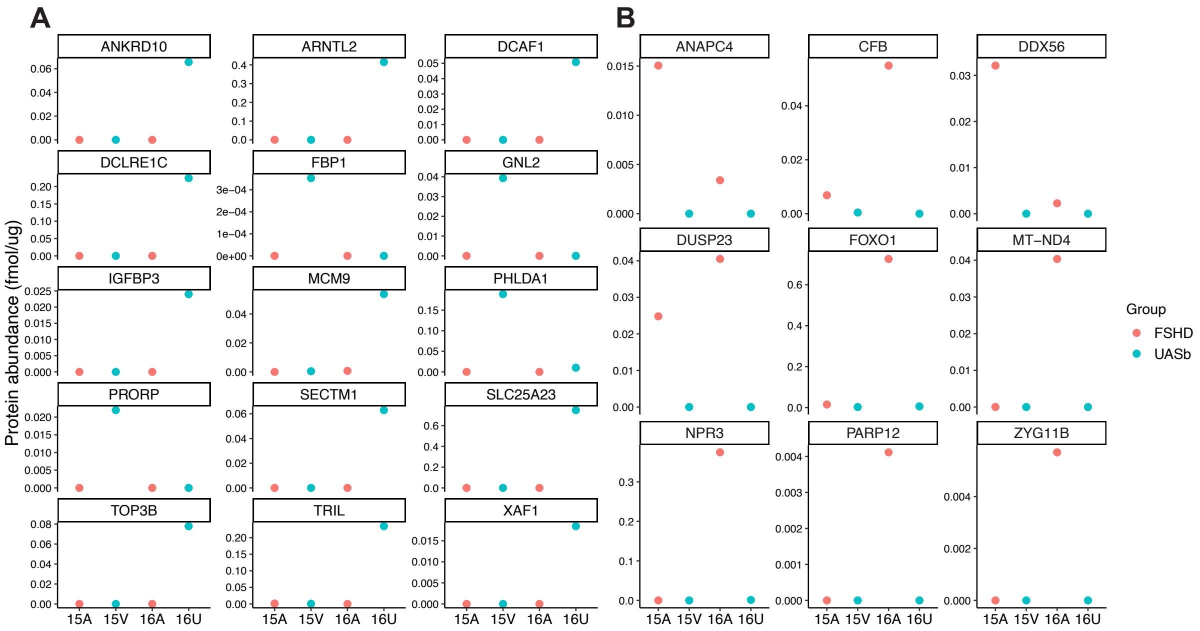

Figure S1: Family and group-specific proteins.

Dot plots reporting the abundance of proteins specifically detected in one family member (**A**) UASb-specific or (**B**) FSHD-specific.

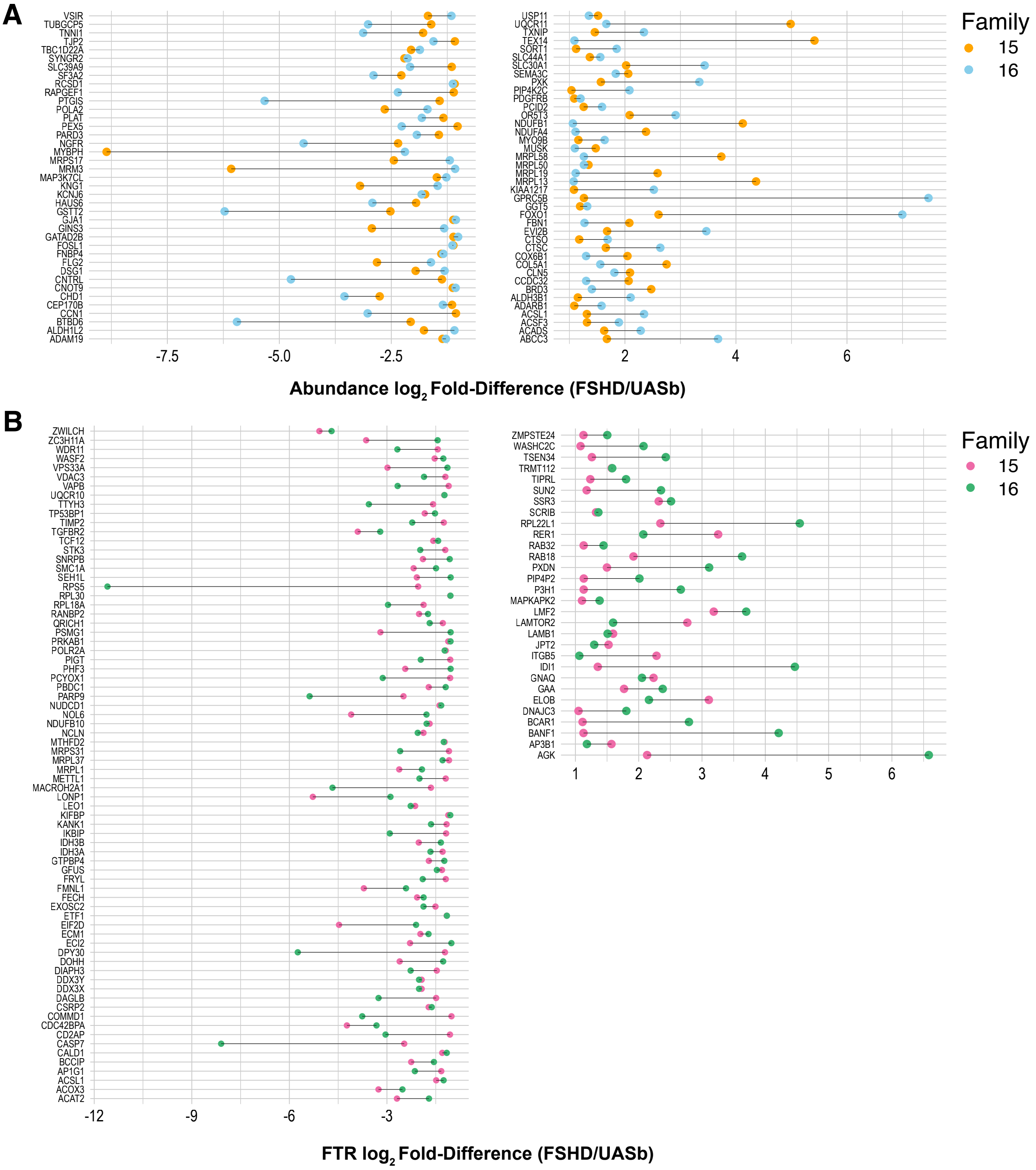

Figure S2: Common proteins that are strongly regulated in FSHD myoblasts in both family pairs.

Data correspond with manuscript figure 5. (**A**) Cleveland dot plot of strongly regulated proteins, including 39 commonly less abundant (log_2 Diff. <-1) or 40 commonly more abundant (log_2 Diff. >1) in FSHD myoblasts. (**B**) Cleveland dot plot of strongly regulated proteins, including 74 commonly slower (log_2 Diff. <-1) or 30 commonly faster (log_2 Diff. >1) turnover proteins in FSHD myoblasts as identified.

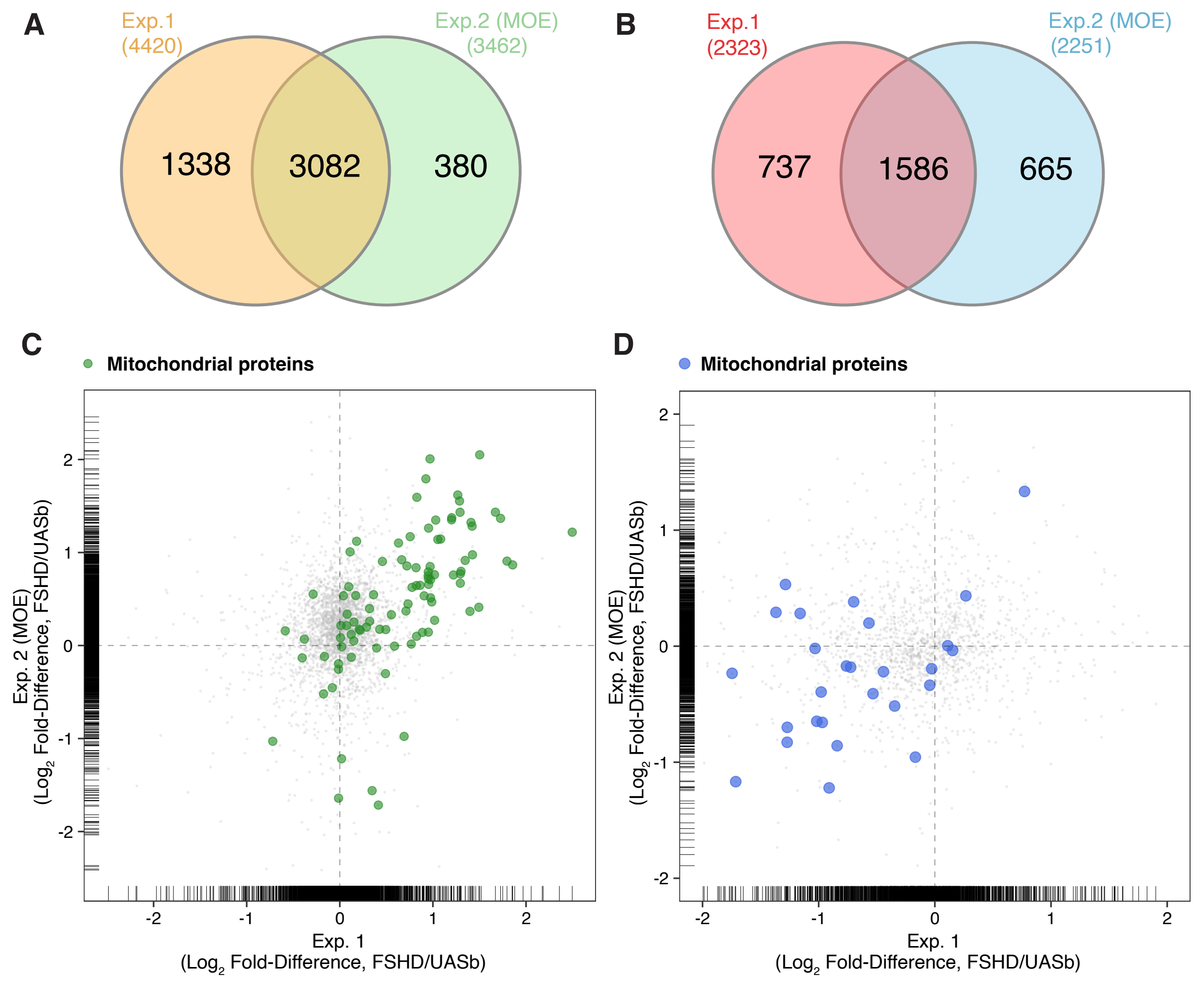

Figure S3: Two independent experiments report mitochondrial proteins are more abundant and have slower FTR in FSHD compared to UASb myoblasts.

Venn diagrams illustrating the extent of overlap in protein abundance data (**A**) and (**B**) FTR data between Experiment 1 and an independent verification experiment (Exp 2), which also included treatment of cells with MOE-antisense oligonucleotide. Scatter plot illustrating the shared patterns of difference in (**C**) protein abundance and (**D**) protein FTR.

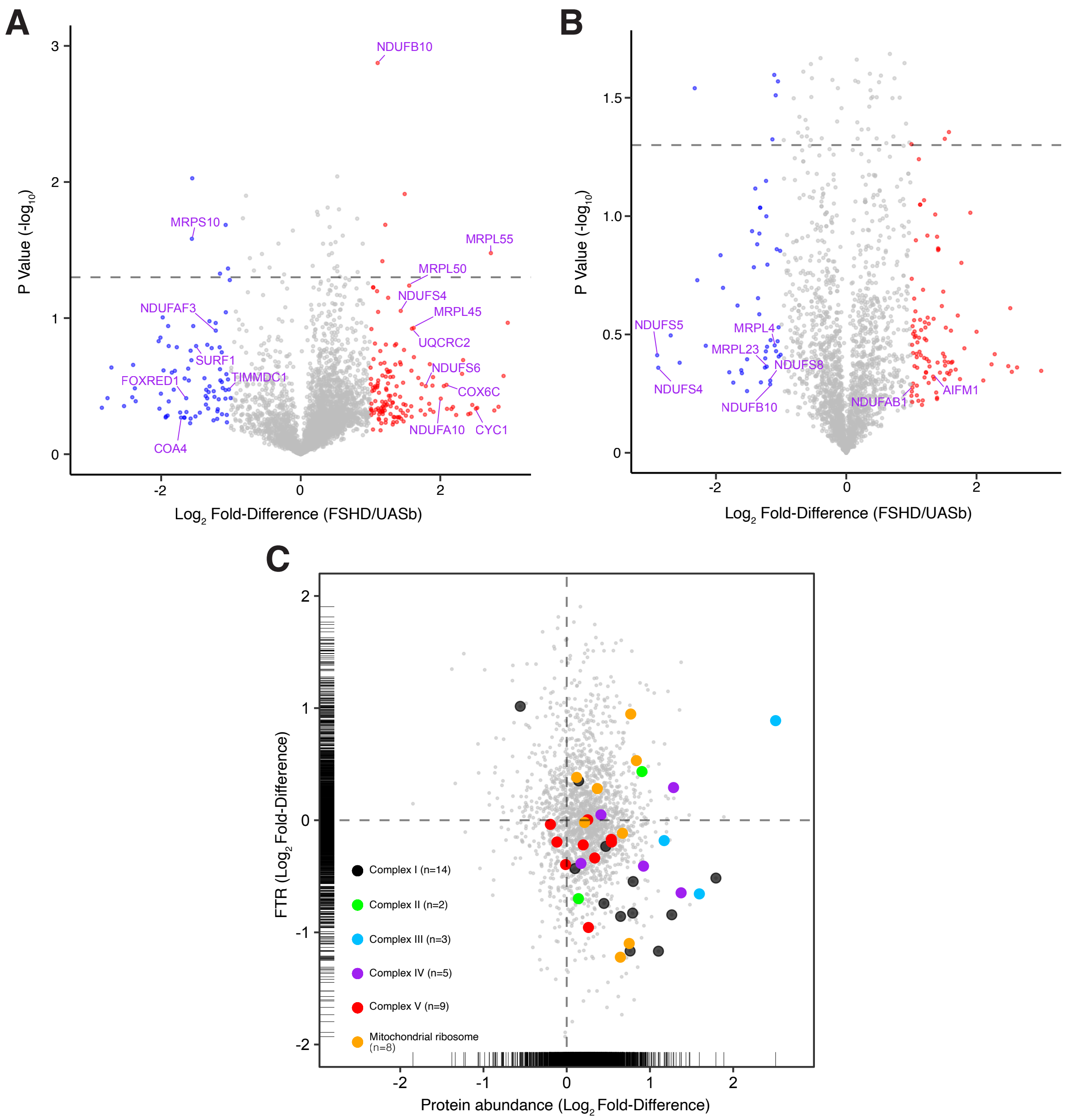

Figure S4: Mitochondrial proteins are more abundant but exhibit slower FTR in FSHD myoblasts as compared to UASb.

Data generated in Exp 2 only and corresponding to manuscript Fig.6. (**A**) Volcano plot comparing the Log_2 Fold-Difference (FSHD/UASb) protein abundance plotted against the -Log_{10} P value (n = 3462). Coloured datapoints represent proteins more abundant (red, log_2 Diff. >1), less abundant (blue, log_2 Diff. <-1), or stable (grey, log_2 Diff. <1 and >-1) in FSHD compared to UASb myoblasts. Dashed horizontal line represents a significance threshold P < 0.05. (**B**) Volcano plot comparing the Log_2 fold-change (FSHD/UASb) FTR plotted against the -Log_{10} P value (n = 2251). Coloured datapoints represent proteins with faster (red, log_2

Diff. 1≥), slower (blue, $\log_2$ Diff. <-1), or stable (grey, $\log_2$ Diff. <1 and >-1) turnover in FSHD compared to UASb myoblasts. Dashed horizontal line shows a threshold of statistical significance ($P < 0.05$). (**C**) Scatter plot comparing the differences in the Log_2 Fold-Difference (FSHD/UASb) between protein abundance and FTR. Rug plots display distribution of individual data both in X and Y axis.

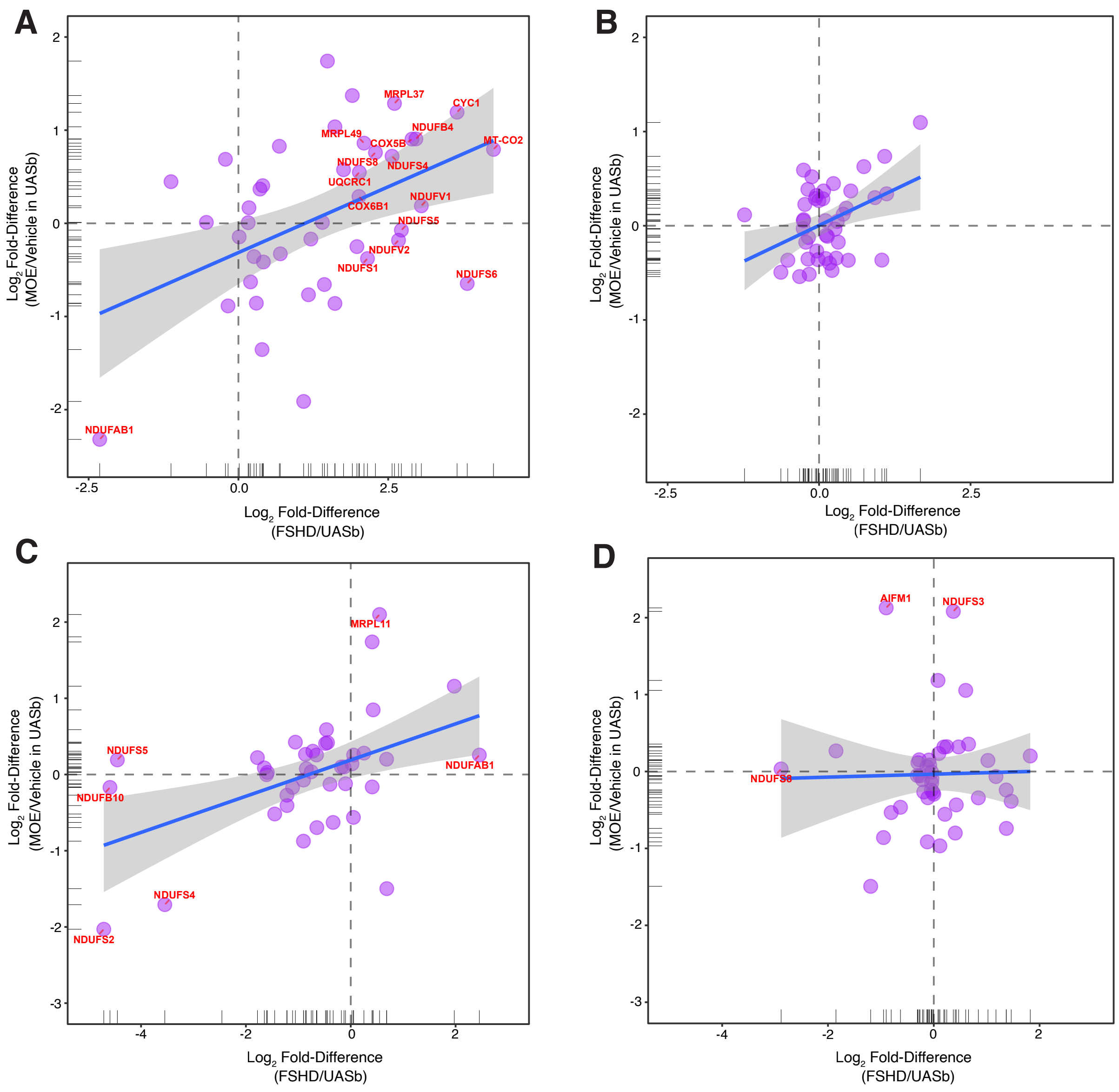

Figure S5: 2’-MOE treatment did not alter mitochondrial protein abundance or FTR in UASb myoblasts.

Scatter plots comparing the Log_2 Fold-Difference between FSHD and UASb (x-axis) and the Log_2 Fold-Difference in 2’-MOE treated UASb myoblasts against vehicle control for abundance data of family #15 (**A**) or family #16 (**B**) and FTR data of family #15 (**C**) or family #16 (**D**). Rug plots display distribution of individual data both in X and Y axis. Linear regression line with 95% confidence interval was drawn in each figure.
